# A method for analyzing censored survival phenotype with gene expression data

**DOI:** 10.1186/1471-2105-9-417

**Published:** 2008-10-06

**Authors:** Tongtong Wu, Wei Sun, Shinsheng Yuan, Chun-Houh Chen, Ker-Chau Li

**Affiliations:** 1 Department of Epidemiology and Biostatistics, University of Maryland, College Park, MD 20742, USA; 2 Department of Biostatistics, Genetics, Carolina Center for Genome Science, University of North Carolina, Chapel Hill, NC 27599, USA; 3 Institute of Statistical Science, Academia Sinica, Taipei 115, Taiwan; 4 Department of Statistics, University of California, Los Angeles, CA, 90095-1554, USA

## Abstract

**Background:**

Survival time is an important clinical trait for many disease studies. Previous works have shown certain relationship between patients' gene expression profiles and survival time. However, due to the censoring effects of survival time and the high dimensionality of gene expression data, effective and unbiased selection of a gene expression signature to predict survival probabilities requires further study.

**Method:**

We propose a method for an integrated study of survival time and gene expression. This method can be summarized as a two-step procedure: in the first step, a moderate number of genes are pre-selected using correlation or liquid association (LA). Imputation and transformation methods are employed for the correlation/LA calculation. In the second step, the dimension of the predictors is further reduced using the modified sliced inverse regression for censored data (censorSIR).

**Results:**

The new method is tested via both simulated and real data. For the real data application, we employed a set of 295 breast cancer patients and found a linear combination of 22 gene expression profiles that are significantly correlated with patients' survival rate.

**Conclusion:**

By an appropriate combination of feature selection and dimension reduction, we find a method of identifying gene expression signatures which is effective for survival prediction.

## Background

The DNA microarray technique allows researchers to simultaneously interrogate the expression levels of all genes in an organism. It has been widely applied in disease studies, such as cancer subtype discovery, cancer/normal sample discrimination, disease gene identification [1-3]. Recently several studies have focused on dissecting the relation between survival time and gene expression [4-7]. One difficulty of these studies is that the survival times are often right-censored. For example, at the ends of the studies, some patients may still be alive. We only know that their survival times are greater than the last follow-up time, but not the exact survival times. Thus, treating these censored survival times as the true life times without adjustment will lead to systematic bias. Another difficulty is that microarray gene expression data are often measured at the full genome scale with tens of thousands of gene expression profiles, while the number of patients under study is relatively small, thereby presenting a difficult variable selection problem.

Many methods of correlating patient survival with gene expression are one-step procedures. Individual genes, gene clusters, or linear combinations of genes are selected by unsupervised or supervised methods. The selected gene expression signatures are then directly used to predict survival probabilities. Unsupervised approaches, such as selecting a small number gene clusters by unsupervised clustering [8], has the disadvantage that survival phenotype information is completely ignored in the feature selection step. Most currently available methods are supervised methods. Nguyen et al. [9] proposed to use the standard partial least square (PLS) method in selecting linear combinations of genes. The survival phenotype is utilized because the PLS method selects linear combinations of genes by maximizing their covariances with the survival time. However, the censoring information has been ignored in this procedure. Li et al. [4] proposed to select linear combinations of genes by a partial Cox regression (PCR) method, which is an extension of PLS method for censoring data. The results of PLS and PCR are linear combinations of thousands of genes, which may be difficult to interpret. Furthermore, the appropriateness of the proportional hazard assumption underlying the Cox regression methodology is not free of challenge. A Bayesian variable selection approach based on the accelerated failure time (AFT) model was introduced by Sha et al. [6], but the performance of this method when the AFT assumption itself is violated has not yet been extensively studied.

Recently, several two-step procedure has been introduced. The first step is the preliminary gene filtering, and the second step is to model the survival time with the pre-selected genes. Li et al. [5] used principal component analysis (PCA) for pre-selection. In the PCA step, linear combinations of genes (called the principal components, PC) are sequentially identified by maximizing the variances explained by the PCs, and a small number of PCs that explain most variances are selected. The second step is to apply SIR to identify some linear combinations of PCs to further reduce the dimensionality. This two-step procedure overcomes the difficulty of handling thousands of genes simultaneously and has a good prediction performance for patients' survival probabilities. However, its performance and interpretability could be improved. First, although PCA can effectively reduce the dimension, it ignores the survival phenotype information. In addition, with principal components selected in the first step, the final results are linear combinations of linear combinations of gene expression profiles, of which the meanings are difficult to interpret. Secondly, although Li et al. [5] used SIR to identify the joint space of life time and censor time, they did not conduct the recovery of the life time space, which should be of the primary interest (see [10] for more details about the distinction between life time space and censor time space). Ma et al. [7] proposed a Lasso (least absolute shrinkage and selection operator, see [11]) type of approach for simultaneous gene selection and parameter estimation based on an additive risk model. In their method, a pre-selection step is used to select gene expression profiles correlated with survival time among those patients without censoring. However, in many real data, censoring rate is often very high. For example, in the real data we study in this paper, more than 70% of the survival times are censored. Therefore ignoring the censored patients in pre-selection step may limit the power of this method.

In this paper, we propose a different two-step procedure to identify a gene signature to predict patients' survival probabilities. In the first step, we use a nonparametric approach to impute the survival probabilities for the censored patients based on the well-known Kaplan Meier estimate. We then use the imputed survival probabilities together with the uncensored survival probabilities to pre-select genes via either the correlation or the liquid association (LA) method [12]. In the second step, we apply the modified SIR for censored data (censorSIR, [10]) to further reduce the dimensionality of the selected genes. CensorSIR found projection directions in life time space without imposing any assumption of the structure model (such as the proportion hazard or AFT) between survival time and gene expression profiles. We can use these projection directions to predict survival probabilities or classify newly diagnosed cancer patients.

The rest of this paper is organized as follows. The method section will be arranged into two parts: preliminary gene screening and dimension reduction – censorSIR. Both simulated and real data will be analyzed to illustrate and evaluate our method in the result section. A discussion section is provided at the end.

## Method

### Preliminary Gene Filtering

For effective use of the current SIR methodology, the number of genes (*G*) need to be much smaller than the number of samples (*N*). It is not even appropriate to allow *G *to be in the same magnitude as *N*. However, for most microarray data, *G *is much larger than *N*. We therefore need to first conduct gene screening to reduce the number of genes. In order to achieve this end, we employ both liquid association (LA) [12,13] and correlation to select candidate genes. From the parsimonious predictive model building point of view, we prefer to start from a smaller gene set as long as a reasonable power of prediction can be obtained. Nevertheless, as pointed out by an anonymous referee, in radiation or carcinogen experiments where tissues are exposed, broad and more global changes of gene expressions are expected across the genome. Therefore, if the sample size is small, our preliminary gene filtering may miss some important genes.

#### Imputation of Survival Probabilities

Both LA and correlation computation cannot be applied directly to survival data due to the presence of censoring. To temper the influence of censoring bias, we propose to apply the following nonparametric imputation method for correcting the right-censored survival time before LA/correlation computation. Let *T*_*i*_, *i *∈ {1,...,*N*} be the survival time for patient *i *and *δ*_*i *_be the censoring indicator such that *δ*_*i *_= 0 indicates censoring and 1 indicates actual death. The imputation procedure can be summarized as:

1. Calculate ${\hat{S}}_{i}$ the Kaplan-Meier estimate of the survival probability [14]. Specifically, ${\hat{S}}_{i}=\prod_{t_{j}\leq t_{i}}\frac{n_{j}-d_{j}}{n_{j}}$ where *n*_*j *_is the number of individuals at-risk just prior to time *t*_*j*_, and *d*_*j *_is the number of deaths at time *t*_*j*_;

2. Impute the survival probability by the predicted conditional median

$${\tilde{S}}_{i}=\{\begin{array}{ll} {\hat{S}}_{i} & \text{if }\delta_{i}=1\\ {\hat{S}}_{i}/2 & \text{if }\delta_{i}=0 \end{array};$$

3. Calculate the percentile $p_{i}=1-{\tilde{S}}_{i}$;

4. Perform the normal quantile transformation on *p*_*i*_.

The normal quantile transformation is necessary for the LA calculation (see next section) and make our procedure robust against outliers. Specifically, it is carried out as follows. For any variable *Z *observed in the *N *patients, we rank all *Z*_*i*_, *i *= 1,...,*N *and denote the rank as *R*_*i*_. The normally transformed profile is then defined as Φ^-1 ^(*R*_*i*_/(*N *+ 1)), where Φ(.) is the cumulative standard normal distribution. Notice that, instead of imputing the survival time, we actually impute the survival probability.

A justification for the above imputation procedure is given next. Suppose that *Z *is the true survival time and its density and survival functions are *f*(*z*) and *S*(*z*) ≡ *P*(*Z *> *z*), respectively. If the squared error loss is used, to predict/impute the true survival time *Z *for a censored patient with censoring time *T*_*i*_(*δ*_*i *_= 0), we can compute the conditional mean given *Z *> *T*_*i*_

$$E(Z|Z>T_{i})=\frac{\int_{z>T_{i}}zf(z)dz}{S(T_{i})}=T_{i}+\frac{\int_{z>T_{i}}S(z)dz}{S(T_{i})}.$$

A natural estimate of *S*(*z*) is to use the Kaplan-Meier estimate of the survival function. However, the resulting estimator is inappropriate if the last observation is censored, because the Kaplan-Meier estimator is undefined beyond the largest uncensored survival time and the integral will be infinite [15]. In practice, for many real data, including the ones we will analyze in this paper, the last observation is censored. Therefore, we will not adopt this conditional mean estimate.

If the absolute value of error is used, we can predict the true survival time *Z *for a censored observation by the conditional median ${\tilde{T}}_{i}=\text{median }(Z|Z>T_{i})$. This means

$$P(Z>{\tilde{T}}_{i})/P(Z>T_{i})=1/2,$$

which leads to

$$S({\tilde{T}}_{i})=S(T_{i})/2.$$

Now we can estimate the survival probability of ${\tilde{T}}_{i}$ by $\hat{S}(T_{i})/2$, where $\hat{S}(t)$ is the Kaplan-Meier estimate of survival function.

It is worth to emphasize that the imputed survival probabilities are only used in the preliminary gene filtering step. The observed survival time with censoring information are used in the dimension reduction step by censorSIR.

#### Liquid Association

LA was originally introduced for studying coexpression patterns between three genes. Specifically, we assume that the correlation of two genes (*X *and *Y*) may vary, depending on the underlying cellular states. For example, *X *and *Y *may be positively correlated at state 1, and negatively correlated at state 2. The overall correlation coefficient could be around zero because the positive and negative correlations might cancel each other out. If the expression of another gene, denoted as *Z*, can reflect the change of cellular state, the correlation between *X *and *Y *can be detected by conditioning on *Z*. Suppose that when *Z *is lowly expressed, *X *and *Y *are positively correlated and when *Z *is highly expressed, *X *and *Y *are negatively correlated. In other words, the increase in the expression of *Z *is associated with the decrease of the correlation between *X *and *Y *. Then the pair (*X, Y*) is called a negative LA pair (LAP) of *Z *and a negative score is assigned. Similarly, if the increase in the expression of *Z *is associated with the increase of the correlation between *X *and *Y*, a positive LA score is assigned. Extreme LA scores, either positive or negative, are of interest. In this context of survival studies, we take the survival probability (after imputation for censored cases) as the third variable to find gene pairs whose coexpression pattern may vary as the survival probability changes. Biologically we expect these genes detected may be associated with molecular pathways related to survival. Therefore we wish to select genes with highest LA scores as candidates for constructing gene signatures to predict survival phenotype.

Based on [12], the LA of *X *and *Y *with respect to *Z*, which measures how the conditional expectation of *XY *given *Z *= *z *varies as *z *varies, is given by

*LA*(*X*, *Y*|*Z*) = *Eg*'(*Z*),

where

*g*(*z*) = *E*(*XY*|*Z *= *z*).

If *Z *follows standard normal distribution, the liquid association can be easily computed by Stein's Lemma [16].

$$\begin{array}{lll} LA(X,Y|Z) & = & Eg^{\prime }(Z)=Eg(Z)Z\\ & = & EE(XY|Z)Z=E(XYZ)\\ & \approx & \frac{\sum_{i=1}^{m}X_{i}Y_{i}Z_{i}}{N}, \end{array}$$

where *N *is the number of samples. Note that due to the normality assumption of *Z*, a normal quantile transformation should be performed before the LA computation [12]. In this study, we normalize both the survival probability and gene expression profiles by normal quantile transformation to ensure the robustness of our method.

#### Feature selection

After imputation of survival probability and normal quantile transformation of both survival probability and gene expression profiles, we can now calculate the correlation between survival probability *Z *and gene expression profile *X*, and the LA score *LA*(*X*, *Y*|*Z*), where *Y *is another gene expression profile. The gene pair (*X, Y*) is chosen from the whole genome, therefore we calculate LA scores for all the *G*^2 ^gene pairs. Both LA and correlation calculation can be conducted in the LA website (). The candidate genes can be selected from both the correlation and LA results. Because of the large number of comparisons in the LA results, the signals may be difficult to detect by examining each individual LA pair (LAP). One alternative strategy is to examine a subset of LAPs with the most extreme LA scores and extract the recurrent patterns of some genes. We refer those recurrent genes as LA hub genes. The effectiveness of this strategy is demonstrated in the Result section.

### Dimension Reduction: SIR and Modified SIR for Censored Data (censorSIR)

We will briefly introduce SIR and censorSIR in this section and discuss the related issues of applying censorSIR to gene expression data. The theoretical derivation and implementation of censorSIR, as well as a simulation example are presented in our supplementary materials. Interested readers are referred to [17] and [10] for more details. An R package of censorSIR is available at .

The original sliced inverse regression (SIR) [17] is a dimension reduction method for regression problems. It reduces the dimension of covariates (denoted as **X**, which is a matrix with each column corresponding to a single covariate) by identifying the projection directions *β*_1_,...,*β*_*k *_in the following model

(1) $$\begin{array}{cc} Y=f({\beta^{\prime }}_{1}X,...,{\beta^{\prime }}_{k}X,\epsilon ), & \begin{array}{cc} \text{with} & {\beta^{\prime }}_{i}\Sigma_{X}\beta_{i}=1, \end{array} \end{array}$$

where *Y *is the response variable, Σ_**X **_is the covariance matrix of **X**, and the random error *ϵ *follows an unknown distribution (we do not need any prior assumption about the distribution of *ϵ *in order to estimate *β*_1_,...,*β*_*k*_) and is independent of **X**. If *f *is known, then equation (1) is not much different from a simple neural network model or a nonlinear regression model. But what makes SIR special is that *β*_1_,...,*β*_*k *_can be estimated while even if *f *is unknown. The space spanned by ${\beta^{\prime }}_{1}X,...,{\beta^{\prime }}_{k}X$, which is a subspace spanned by all the columns in **X**, is called the effective dimension reduction (e.d.r.) space.

SIR can be implemented as follows. First the response variable *Y *is sliced into *h *intervals (SIR is insensitive to the choice of *h *as long as *h *> *k*). Then the inverse mean *E*(*X*|*Y*) is estimated by taking the average of all the values of *X *in each slice, where *X *is one predictor, i.e., one column of **X**. Finally the projection directions *β*_1_,...,*β*_*k *_can be identified as the eigenvectors in the eigenvalue decomposition of the between-slice covariance matrix Σ_**X**|*Y *_= Cov[*E*(**X**|*Y*)] with respect to Σ_**X **_= Cov(**X**) (see [17] for the proof). The number of significant projections, denoted by *k*, can be determined by an asymptotic Chi-square test, which tests the hypotheses *k *= *m *versus *k *> *m *for *m *= 0,...,*p *- 1, where *p *is the number of covariates.

The paper by Li et al. [10] extended the original SIR to censored data. Denote *Y*^0 ^as the underlying true survival time, *C *as the censoring time, and *T *= min{*Y*^0^, *C*} as the observed survival time. Two censoring mechanisms are discussed:

1. *C *is independent of **X **and *Y*^0^;

2. Conditional on **X**, *C *is independent of *Y*^0^.

In the first situation, the general theory of SIR is applicable without modification [10]. However, the second censoring mechanism, which is more common, will introduce bias to the SIR estimations. To address this question, Li et al. [10] introduced a two-step procedure. The basic idea is to estimate a weight function *ω*(*Y*, *t*, **X**) for the patient with censoring time *Y *and covariate **X **at time *t *with *t *> *Y*. Specifically, *ω*(*Y*, *t*, **X**) = *S*^0^(*t*|**X**)/*S*^0^(*Y|***X**), and *S*^0^(*t*|**X**) = *P*(*Y*^0 ^≥ *t*|**X**). The weight function can be estimated by any kernel method. Effective dimension reduction (e.d.r.) space of life time can be identified given this weight function. Because kernel estimation is more efficient in low-dimension spaces, one initial dimension reduction step is required. Assume that the true underlying survival time *Y*^0 ^and the censoring time *C *have dimension reduction structures given by

(2) $$Y^{0}=g({\beta^{\prime }}_{1}X,...,{\beta^{\prime }}_{k}X,\epsilon )$$

and

(3) $$C=h({\gamma^{\prime }}_{1}X,...,{\gamma^{\prime }}_{c}X,\epsilon^{\prime }),$$

respectively. First, the uncensored observations and the censored observations will be sliced separately, namely the double slicing procedure. Then the joint e.d.r. space of the underlying survival time and censoring time will be obtained by taking the eigenvalue decomposition on the between-slice covariance matrix with respect to the covariance matrix of **X**. The leading eigenvectors will serve as the projection directions

After identifying the gene expression signatures (the projection directions), one can use scatter plots (2d or 3d) or non-parametric fittings (e.g., splines) to explore the possible forms of the function *g *in equation (2). Predictive model can also be build based on the reduced expression data. For example, survival models, e.g., Cox proportional hazard model, can be fitted with the projected directions as the explanatory variables first and then the fitted model can be used to predict the survival probability of a newly diagnosed patient. These gene expression signatures can also be used to classify the cancer patients into different treatment groups for better clinical outcomes.

## Results and Discussion

### Simulation

Clustering is an important feature in gene expression data. Genes involved in the same or related biological process are likely to coexpress, so that the expression profiles of these genes form a cluster. Thus besides simulating independent expression profiles, we also simulate gene expression clusters. Specifically, we simulate four clusters with the corresponding gene indexes as: (11–15), (16–20), (3, 31–34), (4, 41–44). In each cluster, each vector *X*_*i *_∈ *R*^*p *^of gene expression is simulated from a multivariate normal distribution whose marginal distributions are standard normal, and the five genes are correlated with each other with a common correlation coefficient *ρ*. The rest of the genes are uncorrelated and are simulated by standard normal distribution. The true survival time is generated by

*Y*^0 ^= exp (*W*_1_*β*_1 _+ *W*_2_*β*_2 _+ *X*_3_*β*_3 _+ *X*_4_*β*_4_),

where *β*_*k *_= 0.5 for *k *= 1,...,4 and

*W*_1 _= (0.15*X*_11 _+ 0.3*X*_12 _+ 0.45*X*_13 _+ 0.6*X*_14 _+ 0.75*X*_15 _+ 0.5) · *X*_1_

*W*_2 _= (0.15*X*_16 _+ 0.3*X*_17 _+ 0.45*X*_18 _+ 0.6*X*_19 _+ 0.75*X*_20 _+ 0.5) · *X*_2_,

The censoring time is generated by

*C *= exp (*X*_6_*γ*_1 _+ *X*_7_*γ*_2 _+ *X*_8_*γ*_3 _+ *X*_10_*γ*_5_),

where *γ_l _*= 0.1 for *l *= 1,...,5. The observed survival time is the minimum of *Y*^0 ^and *C*. With *n *= 500, *p *= 10, 000, we generate 50 random samples for *ρ *= 0.4, and 0.8, representing the modest and high correlation cases respectively. The censoring rate is about 50% in both cases.

In this setup, the two genes *X*_1 _and *X*_2 _act as the LA hub genes. We analyze the simulated data in two steps. In step one, survival probabilities are imputed and normalized by normal quantile transformation and candidate genes are selected based on the LA and the correlation scores. Genes that appear at least three times in top 50 positive and negative LA pairs are selected. The first 10 genes with the highest correlations with survival probability in absolute values are also selected. Table 1 reports the average number of genes selected from LA (*n*_1_) and correlation (*n*_2_), the average number of correct genes *K*_*true*,1 _and *K*_*true*,2 _selected, and the average number of predictors *K*_*cluster*,2 _found in the clusters 31 – 34 and 41 – 44. We took *ρ *= 0.4 and 0.8 to represent modest and high co-regulation within a cluster of genes. The LA hub genes *X*_1 _and *X*_2 _can be found by LA every time. Correlation also works well since the genes in the same clusters of *X*_3 _and *X*_4 _were also picked up.

**Table 1:** Average numbers of selected and true predictors found in simulation

*ρ*	*n*_1_	*K*_*true*,1_	*n*_2_	*K*_*true*,2_	*K*_*cluster*,2_
0.8	3.56	2	10	2	7.22
0.4	2.54	2	10	2	2.6

This table reports the average numbers of genes selected from LA (*n*_1_) and correlation (*n*_2_), the average numbers of correct genes selected from LA (*K*_*true*,1_) and correlation (*K*_*true*,2_), and the average numbers of predictors *K*_*cluster*,2 _found in the clusters 31 – 34 and 41 – 44 for *ρ *= 0.8 and 0.4. The sample size is (*n*, *p*) = (500,10000).

In step two, the dimension reduction is performed on the selected genes, followed by using the first SIR component as the gene signature score for survival prediction. To test the effectiveness of gene signature, we want to test if the gene signature can separate the patients into high and low risk groups. We define the cutoff as the median of the gene signature, i.e.

$$\tilde{x}=X^{\prime }b,$$

where **X **is the matrix of genes selected from step one, and *b *is the first SIR direction. A testing data set of 1,000 subjects are generated similarly as the training data, we divide the 1,000 subjects into higher and lower groups based on whether the score $\tilde{x}$ is higher or lower than the cutoff and apply the log rank test. The simulation result shows that the p-value of the log rank test is smaller than 10^-22 ^in every simulation run. Table 2 reports the quantiles of the *p*-values (in *log*_10 _scale).

**Table 2:** Quantiles of the *p*-values (in *log*_10 _scale) of the log rank test for testing data

*ρ*	0%	25%	50%	75%	100%
0.8	-48.6007	-41.2775	-36.3042	-28.5859	-22.0066
0.4	-52.4528	-45.4521	-38.5598	-33.7795	-22.1615

Table 3 reports the average absolute coefficients in the first SIR direction for (1) true underlying genes 1 to 4; (2) the genes in the clusters 31–34 and 41–44; and (3) all the other genes. The genes *X*_3 _and *X*_4 _have the highest coeffcients since they have high first-order correlations with the survival time. The coefficients of the LA hub genes *X*_1 _and *X*_2 _are slightly lower than the coefficients of *X*_3 _and *X*_4_, but higher than ones of noise genes. In other words, censorSIR further identifies the true genes by showing higher weights in the projection direction.

**Table 3:** Coefficients in the censorSIR projection direction in simulation

*ρ*	*X*_1_	*X*_2_	*X*_3_	*X*_4_	Cluster	Other
0.8	0.2441	0.2299	0.6222	0.5825	0.1321	0.1133
0.4	0.2200	0.2331	0.5347	0.5240	0.0620	0.1591

This table reports the average absolute values of the coefficients for genes 1–4, genes in the clusters (31–34) and (41–44), and noise genes in censorSIR directions.

### Application in NKI breast cancer data

In this section, we present the results of analyzing the NKI breast cancer dataset [18] using our method. The data can be downloaded from . Because this data set was initially generated by the Netherlands Cancer Institute (Nederlands Kanker Instituut in Dutch, NKI), it is referred to as NKI breast cancer data or NKI-295. Seventy-nine out of the 295 patients died before the study ended, which yields 79 real survival times and a heavy censoring rate of 73.2%. The expression levels of 24,481 genes were measured for each of the 295 patients.

#### Gene Signature Identification

Following the steps described in the Method section, we first impute survival probabilities and normalize the imputed survival probabilities and gene expression profiles by normal quantile transformation. Assigning the processed survival time as *Z*, we apply LA with all the 24,481 genes serving as *X *and *Y *to calculate LA score *LA*(*X*, *Y*|*Z*). We select 11 genes that appear at least 3 times in the top 50 (positive) or bottom 50 (negative) LA pairs, as well as 11 genes of which the absolute value of the correlation coefficient with the processed survival time is bigger than 0.3 (Table 4). These cutoffs are determined based on the results of 1000 permutations. In each permutation, we permute the survival time and censoring time of the 295 patients simultaneously, and then calculate the LA scores and correlation scores as for un-permuted data. We use the number of reoccurrences of one gene in top 50 or bottom 50 LA pairs to evaluate the importance of that gene. A threshold of three reoccurrences is used to select LA hub genes. Our simulation tells how many LA hub genes can occur by chance alone. It turns out that among the 1000 permutations, on the average there are only four LA hub genes present, as compared to the 11 LA hub genes detected in the real data. To evaluate the significance of correlation score, we record the highest correlation (absolute value) between the processed survival time and all the 24,481 gene expression profiles. In only 3 out of the 1000 permutations, we observe a correlation score greater than 0.3 (absolute value). The permutation results for both LA and correlation scores indicate that at least the majority of the genes selected are significantly related with survival time after the genome-wise multiple testing correction. It is possible to select more genes based on the correlation criterion, but we prefer a parsimonious model to serve the purpose of prediction and avoid overfitting.

**Table 4:** Twenty-two genes selected by LA and correlations

**Official Symbol**	**Official Name**	**Annotation**
ABCG1 (3)	ATP-binding cassette, sub-family G (WHITE), member 1	ATP binding; cholesterol homeostasis

BIRC5 (-0.31)	baculoviral IAP repeat-containing 5 (survivin)	Colorectal cancer; apoptosis

C5orf30 (3)	chromosome 5 open reading frame 30	

CENPA (-0.32)	centromere protein A	chromosome organization and biogenesis

CTSL2 (-0.33)	cathepsin L2	cathepsin L activity; proteolysis

E2F7 (-0.31)	E2F transcription factor 7	breast cancer cell growth [30].

ERBB2 (3)	v-erb-b2 erythroblastic leukemia viral oncogene homolog 2	member of the epidermal growth factor (EGF) receptor family of receptor tyrosine kinases; Amplification and/or overexpression in numerous cancers, including breast and ovarian tumors

FAM150B (3)	family with sequence similarity 150, member B	

H06509 (3)	mRNA sequence	

HJURP (-0.31)	Holliday junction recognition protein	up-regulated in lung cancer

KIF20A (-0.30)	kinesin family member 20A	Collaboration of KIF20A and disc large homologue 5 is likely to be involved in pancre atic cancer [31]

KIFC1 (-0.30)	kinesin family member C1	mitotic sister chromatid segregation

KRT6B (4)	keratin 6B	Cell Communication; ectoderm development

LOC284072 (3)	hypothetical protein	

ORMDL2 (4)	ORM1 (S. cerevisiae)-like 2	expressed in normal aorta

PDGFRA (4)	platelet-derived growth factor receptor, alpha polypeptide	Prostate cancer; cell proliferation

PELI1 (3)	pellino homolog 1 (Drosophila)	role in interleukin-1-mediated signaling through interaction with interleukin-1 receptor-associated kinase 4-IRAK-tumor necrosis factor receptor-associated factor 6 complex [32]

PERLD1 (5)	per1-like domain containing 1	gastric cancer [33]

PRR11 (-0.32)	proline rich 11	interact with E2F1, E2F4

PTTG2 (-0.33)	pituitary tumor-transforming 2	chromosome organization and biogenesis

QSOX2 (-0.33)	quiescin Q6 sulfhydryl oxidase 2	oxidoreductase activity; cell redox homeostasis

TROAP (-0.32)	trophinin associated protein (tastin)	cell adhesion

The numbers in the parenthesis are either the number of times the gene appear in top/bottom 50 LA pairs or the correlation coefficient. Only genes with negative correlations are selected because positive correlations have smaller absolute value. The highest positive correlation is 0.2875, which is ranked as 31st by absolute values.

**Figure 1:**
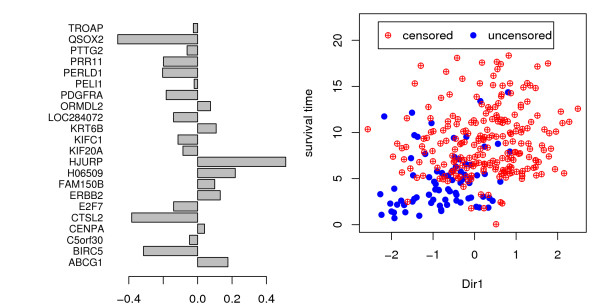
**Underlying direction revealed by censorSIR**. The first projection direction identified by censor SIR for 22 gene expression profiles versus survival time. The left panel shows the projection weights on each of the 22 gene expression profiles, i.e., the eigenvector corresponding to the biggest eigenvalue. Notice we normalize the eigenvector *β*_*i *_so that ${\beta^{\prime }}_{i}\Sigma_{X}\beta_{i}$ is equal to 1, i.e. $Var({\beta^{\prime }}_{i}X)=1$ The right panel shows the scatter plot between projection direction and survival time.

The next step is to perform censorSIR on the 22 selected genes. First, the double-slicing procedure is used to find the joint e.d.r. space of the true survival time *Y*^0 ^and the censoring time *C *[see Section 3 in additional file 1 for more details of the double-slicing outputs]. The *χ*^2 ^test shows that the first projection direction is significant (*p *= 0.000144), and the second direction is mildly significant (*p *= 0.066), while the other directions are not significant (*p *> 0.30). Therefore, we take the first two eigenvectors as the projection directions in the joint e.d.r. space and continue to recover the e.d.r. space of true survival time *Y*^0^. Now with only the first eigenvalue being significant (*p *= 7.66*e*^-5^), we have reduced the survival time space to one dimension (Figure 1) [see Section 3 in additional file 1 for details of the censorSIR outputs]. In the following discussion, we only consider the projection of gene expression data in the first projection direction: $\tilde{x}={\beta^{\prime }}_{1}X$

We fit a Cox's model with the covariate $\tilde{x}$ such that

$$h(t|\tilde{x})=h_{0}(t)\exp(a\tilde{x})$$

The estimated coefficient $\hat{a}$ is -0.93 (p-value = 2.2*e*^-16^), with 95% confidence interval of hazard rate [0.313, 0.496]. From the coefficients of the 22 genes in the e.d.r. direction, i.e., *β*_1_, we can see that both genes recruited via correlation and liquid association can have large impacts on the projection direction (Figure 1). Both positive and negative coefficients are observed. Since the projection is positively correlated with survival time, genes with negative coefficients are possible "oncogenes", meaning higher expression is associated with higher risk. In contrast, genes with positive coefficients are possible "tumor repressor genes", meaning higher expression is associated with lower risk. We also use Cox's model to test the efficacy of using each single gene of the 22 genes to predict the survival probability. The most significant p-value that can be achieved by a single gene is 4*e*^-11^, which is significant, but much less significant than the result using their linear combination.

#### Prediction and Cross-validation

To predict the survival probability of a newly diagnosed breast cancer patient, we will only need to check the expression levels of the 22 genes and then compute $\tilde{x}$. The survival probability of this patient can be easily predicted using the formula

$$S(t|x)=\exp\left(-\int_{0}^{t}h_{0}(u)e^{-0.93\tilde{x}}du\right).$$

The baseline hazard function *h*_0_(*t*) can be estimated by the Breslow estimators after the coefficients of the Cox's model have been estimated [15,19,20]. Other methods, including the exact method [21], the discrete method [22] and the Efron's method [23], can handle observations that have tied survival times. Most statistical software provides the baseline cumulative hazard function, for example, the function "basehaz" in R.

**Figure 2:**
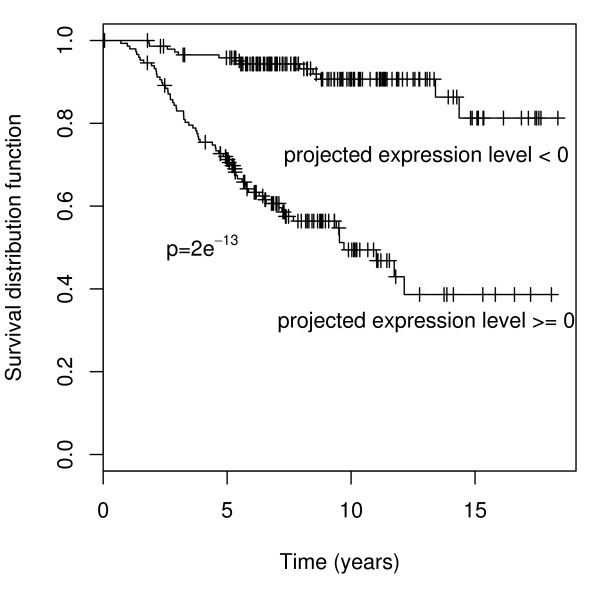
**The Kaplan-Meier estimates of survival rates**. Survival rates are estimated for the two groups of patients of sizes 148 and 147 based on the expression of the selected gene signature. The log-rank test comparing the two curves gives a p-value of 2*e *- 13.

We divide the patients into two groups of approximately equal sizes (148 vs. 147) based on the gene expression level ($\tilde{x}$). The log-rank test shows that the survival rates of these two groups are significantly different (*p *= 2*e*^-13^, Figure 2). Here a note of caution in interpreting the face value of this significance level is important. As suggested by one referee, it is instructive to construct a null situation where the survival data and the gene expression data are completely unlinked. We took this suggestion by first randomly permuting the gene expression data and then carrying out the same steps of our gene signature procedure for correlating the permuted gene expression data with the un-permuted survival data. It turns out that after the steps of candidate gene selection, the SIR analysis does not detect any significant direction (*p *= 0.45) [see Section 3 in additional file 1 for details of the censorSIR outputs]. This is the correct conclusion and if this was the real life situation, we should stop here. We would not recommend using the first SIR direction for constructing gene signature and splitting the patients into high and low risk groups. However, if one ignored the SIR significance test and went on to split the patients, then a statistically significant separation was observed with a significant p-value of *p *= 6*e*^-6 ^[see Section 3 in additional file 1 for a plot of the results]. This artifact is largely due to the smallness of the sample size which leads to the chance of overfitting in the permuted data, a phenomenon similar to the one commonly faced in multiple testing without adjustment. This result speaks for the needs of conducting the SIR significance test. In the real data we did find that the first SIR direction has a significant p-value (*p *= 7.66*e *- 05 versus *p *= 0.45) and the p-value for the log rank test in comparing high and low risk groups is also much lower (*p *= 2*e*^-13 ^versus *p *= 6*e*^-6^).

To assess the prediction ability of our method, we carry out 100 cross-validations. In each cross-validation, we randomly divide the 295 patients into training and testing data of sizes 148 and 147, respectively. Only the training data are used to identify the gene signatures (from survival time imputation to the censorSIR), and then the identified gene signature is tested in the testing data. Based on the training data, genes appear at least three times in the top/bottom 50 LAPs are selected. The median number of genes selected via LA is six with 1st/3rd quantiles as four and eight, respectively. In addition, 10 genes that have the highest absolute correlation with the processed survival time are also selected. Five out of 100 cross-validations have no significant e.d.r. directions (*p *> 0.05). For the rest 95 cases, we conduct the log-rank test to test how well the most significant SIR directions differentiate the survival rates in both training and testing data. For the training data, the median, 1st, and 3rd quantile of log-rank p-values are 4.0*e*^-10^, 3.2*e*^-11^, and 1.0*e*^-8^, respectively. For the testing data, the median, 1st, and 3rd quantile of log-rank p-values are 4.4*e*^-3^, 7.2*e*^-4^, and 1.4*e*^-2^, respectively. Among the 95 cross-validations, 84 of them yield a testing p-value smaller than 0.05. We conclude that our method has a reasonably good prediction power even for such a small training sample with a high censoring rate.

## Discussion

In this paper, we have introduced a two-step method for the joint analysis of survival time and gene expression data. The first step is the pre-selection of gene expression profiles. In order to offset the bias of the censored survival time, we employ a nonparametric method to impute the censored survival time. This method is simple to implement but we agree with an anonymous referee that future improvement would be desirable. Both correlation and LA are then used as the criteria to pre-select genes related with the imputed survival time. In the second step, with these selected genes, the modified SIR for censored data is conducted to further reduce the dimension of the gene expression data by identifying a few projection directions. Two major advantages of censorSIR over other methods are: 1) it employs the information of both survival time and gene expressions; 2) it does not require any function form for the relation between survival time and the projection directions. No explicit parametric assumptions are needed in the whole dimension reduction procedure.

A data set of 295 breast cancer patients was analyzed using the proposed method. A single projection direction (linear combination of 22 gene expression profiles) was identified that is significantly related to the survival time. Several studies have been done on this data set ([18,24-26]). One gene signature of 70 genes is identified by maximizing the accuracy of classifying the patient with distant metastases within 5 years or not [24]. van de Vijver et al. [18] showed that this signature of 70 genes can also be used to predict the survival probabilities of the 295 patients. Chang et al. [25] employed 442 "core serum response" (CSR) genes for the survival probability prediction. Perou et al. [2,27] have used clustering strategy to identify a set of 1410 "intrinsic genes", which can be used to classify patients into five subtypes. Each subtype has different levels of risk, therefore can be related with survival time [26]. Although these three gene sets (70-gene, CSR genes, intrinsic genes) only overlap slightly, they yield similar prediction strength, which leads to the conclusion that overlaps between gene expression signatures might not be a necessary measure of reproducibility [26]. Majority of the 22 genes we identified have not been included in these three gene sets. Nevertheless, the high accuracy of classifying patients in terms of their survival probabilities by only 22 genes demonstrates the efficacy of our method.

We have conducted simulation studies to demonstrate the effectiveness of our method. Our simulation setting intends to address the dependence issue between gene profiles. As the results have shown, it is possible that correlated genes would be selected as the surrogated genes for obtaining gene signatures. This is biologically meaningful because biologically correlated genes are likely to participate in the same pathways. The LA hub genes represent higher order interaction structures which would not be easy to detect by correlation method; see [13] for an illustration in multiple sclerosis candidate gene finding.

One subtle question is how to choose the cutoffs of correlation and liquid association in the pre-selection step. This would surely depend on the size of dataset. The permutation test of correlations/LA scores as developed before can be applied. On the other hand, because SIR requires the inverting of the covariance matrix. Although there are many on-going studies to sidestep this difficulties (for example [28,29]), like most model or variable section procedures, their validity should be taken with caution because some user-specified tuning parameters are always hidden. Our preference is to preset a relatively smaller number of genes (so as to assure the stability of covariance estimation) to begin with. This can be coupled with the procedure of cross-validation to circumvent the overfitting problem to some extent. On the other hand, biological network affecting the survival phenotype is complex and is likely to involve multiple pathways. Thus practically it is unlikely for any method to claim the ability of identifying all genes involved in the survival network. An effective gene signature can still be obtained without having to find every single gene in generating the survival time.

## Conclusion

In summary, we propose an effective dimension reduction and variable selection method to dissect the relationship between gene expression profiles and survival times. Compared with most available methods, our method has two major advantages. Firstly, not only the correlation but also the dynamic correlation between gene expression and survival time are explored by employing liquid association (LA) as part of the feature selection criteria; Secondly, no model assumption is required on the relationship between gene expression and survival time.

## Authors' contributions

Wu and Sun shared equally as the co-first author, with ordering by randomization. Design of the study, problem conceptualization, and envision of statistical methods were contributed by Li, Wu and Sun. Implementation of methods were contributed by Sun and Wu. Liquid association computing is facilitated by an on-line computing system designed by Yuan and Li. Development of Censor-SIR methodology and programming were contributed by Chen and Li. Writing was contributed by Wu, Sun and Li.

## Acknowledgements

Authors would like to thank Dr. Charles Perou and Mr. Cheng Fan for helpful discussions. Research conducted by Li is supported in part by NSF grants DMS0201005, DMS0406091, and DMS-0707160. Li and Yuan were also supported in part by MIB, Institute of Statistical Science, Academia Sinica and grant NSC95-3114-P-002-005-Y and NSC97-2627-P-001-003. Research conducted by Sun is supported in part by NIEHS grant 5 P42 ES05948-15 and 5 P30 ES10126-07.

## Supplementary Material

Additional file 1 **Supplementary Materials**. Method and implementation of censorSIR and additional real data analysis results. Click here for file
