# Supplementary material for: A method for analyzing censored survival phenotype with gene expression data
Source: BMC Bioinformatics. 2008 Oct 6;9:417. doi: 10.1186/1471-2105-9-417 (PMC2579309; doi:10.1186/1471-2105-9-417)
Supplement: Additional file 1 — Supplementary Materials. Method and implementation of censorSIR and additional real data analysis results. [file 1471-2105-9-417-S1.pdf]

# Supplementary Materials: censorSIR

Tong Tong Wu, Wei Sun, Shinsheng Yuan, Chun-Houh Chen, Ker-Chau Li

October 1, 2008

## 1 A brief description of censorSIR

Use $Y$=min($Y^0$, $C$) to denote the observed survival time, where $Y^0$ is the true survival time and $C$ is the censoring time. The $p$ regressors are denoted as $X$. We assume that $Y^0$ has a dimension reduction structure:

$$Y^0 = g(X'\beta_1, ..., X'\beta_k, \epsilon), \tag{1.1}$$

where the functional form of $g$ and the distribution of $\epsilon$ are usually unspecified. Then the e.d.r. space of $Y^0$ can be found by the eigen-value decomposition (1)

$$\Sigma_{\eta^0} b_i = \lambda_i \Sigma_X b_i, \tag{1.2}$$

where $\Sigma_{\eta^0} = cov(E(X|Y^0))$, $\Sigma_X = cov(X)$, and $\eta^0 = E(X|Y^0)$ is the inverse regression curve of survival time. A mathematical proof of finding e.d.r. space by equation (1.2) can be found in Lemma 3.1 of Li (1991). In survival data, equation (1.2) cannot be used directly because the true survival time $Y^0$ is unobservable due to censoring, and thus $\eta^0$ is unknown.

First, if $C$ is independent of $Y^0$ and $X$, then it is easy to show that $\eta = E(X|Y)$, the inverse regression curve of observations $Y$, spans the same

space as $\eta^0 = E(X|Y^0)$ does:

$$E(X|Y) = E(E(X|Y^0, C)|Y) = E(E(X|Y^0)|Y). \tag{1.3}$$

Following equation (1.3), the e.d.r. space of $Y^0$ can be found by the eigenvalue decomposition

$$\Sigma_\eta b_i = \lambda_i \Sigma_X b_i, \tag{1.4}$$

where $\Sigma_\eta = cov(E(X|Y))$, $\Sigma_X = cov(X)$.

In the more general situation where $C$ is only independent of $Y^0$ given $X$, SIR cannot be applied to survival data directly. However, the inverse regression curve can be estimated by

$$\begin{aligned} m_j = E\{X|Y^0 \in [t_j, t_{j+1})\} &= \frac{E\{XI(Y^0 \in [t_j, t_{j+1}))\}}{P\{Y^0 \in [t_j, t_{j+1})\}} \\ &= \frac{E\{XI(Y^0 \geqslant t_j)\} - E\{XI(Y^0 \geqslant t_{j+1})\}}{E\{I(Y^0 \geqslant t_j)\} - E\{I(Y^0 \geqslant t_{j+1})\}}, \end{aligned} \tag{1.5}$$

where $m_j$ is the slice mean of the $j$-th slice. $I(\cdot)$ is the indicator function, and $0 = t_1 < t_2 < \cdots < t_H < \infty = t_{H+1}$ is a partition of the survival time. Replacing $Y^0$ in equation (1.5) by $Y$ and the censoring indicator $\delta$ ($\delta = 0$ if censoring; $\delta = 1$ otherwise) yields the following two equations:

$$\begin{aligned} E\{XI(Y^0 \geqslant t_j)\} = E\{XI(Y \geqslant t_j)\} + \\ E\{XI(Y < t, \delta = 0)\omega(Y, t, X)\}, \end{aligned} \tag{1.6}$$

$$\begin{aligned} E\{I(Y^0 \geqslant t_j)\} = E\{I(Y \geqslant t_j)\} + \\ E\{I(Y < t, \delta = 0)\omega(Y, t, X)\}, \end{aligned} \tag{1.7}$$

where $\omega(Y, t, X) = \frac{S^0(t|X)}{S^0(Y|X)}$ is the weight function, and $S^0(t|X) = P(Y^0 \geqslant t|X)$. The weight function can be estimated by kernel method. The estimator is consistent and it converges at root $n$ rate(1) . Because kernel

estimation is more efficient in low-dimension spaces, one dimension reduction step is required. Similar to equation (1.1), we assume that $C$ also has a dimension reduction structure:

$$C = h(X'\gamma_1, ..., X'\gamma_k, \epsilon'). \tag{1.8}$$

Based on assumptions in equation (1.1) and equation (1.8), the joint e.d.r space of life time and censoring time can be estimated by SIR with double-splicing. In particular, slices are constructed for $\delta = 0$ and $\delta = 1$ separately and then pooled together to estimate the covariance matrix of the inverse regression curve. Suppose $B = (b_1, b_2, ..., b_r)$ are the $r$ eigenvectors spanning the joint e.d.r. space, then the projection of $X$ in the joint e.d.r space is $X'B$. Use $X'B$ as a replacement of $X$ to yield a reliable kernel estimation of the weight function: $\hat{\omega}(t', t, X)$. Then, with $\hat{\omega}(t', t, X)$, the slice mean $m_j$ can be estimated by equation (1.5-1.7). The final estimate of the covariance matrix of the inverse regression curve is:

$$\hat{\Sigma}_{\eta 0} = \sum_j (\hat{m}_j - \bar{X})(\hat{m}_j - \bar{X})'\hat{p}_j, \tag{1.9}$$

where $\hat{p}_j = \hat{P}\{Y^0 \geqslant t_j\} - P\{Y^0 \geqslant t_{j+1}\}$. The eigenvalue decomposition

$$\hat{\Sigma}_{\eta^0} \hat{b}_i^0 = \hat{\lambda}_i \hat{\Sigma}_X \hat{b}_i^0 \tag{1.10}$$

gives the e.d.r. space of life time.

## 2 Implementation of censorSIR

We implemented censorSIR algorithm into an R package: censorSIR, which can be downloaded at http://www.bios.unc.edu/∼wsun/software.htm. Here we discuss some details of implementation and one simulation example.

1. Double-slice the survival time and censoring time to find SIR directions.

2. Find the number of significant SIR directions. The print out of the double slicing result shows the size of eigen-values and the $\chi^2$ test of the SIR directions (Figure 1).

3. The projection of the regressors in the joint e.d.r. space is used to estimate the kernel matrix $M_{n\times n}$, $M[i,j] = K_p(h_n^{-1}(X_j - X_i))$. A Gaussian kernel is used.

4. The conditional survival function is estimated for the $n$ individuals based on the kernel matrix. The weight function and inverse regression function are estimated.

5. Use inverse regression function to estimate covariance matrix and then conduct eigenvalue decomposition to find e.d.r space of lifetime.

The first step is implemented in function *double.slice*. The second step needs user input. The last three steps are implemented in function *censor.sir*. The SIR directions found by double.slice and cen.sir can be plotted in a 2D or 3D space. We use example 4.1 of (1) to demonstrate the usage of this R package. Six regressors $\{x_1, ..., x_6\}$ are generated from a standard

```
Eigenvalues:
               Dir1   Dir2    Dir3    Dir4    Dir5     Dir6
Eigenvalues 0.7783 0.2531 0.03267 0.02664 0.01784 0.007505
Cum.Sum.R^2 0.6974 0.9241 0.95342 0.97729 0.99328 1.000000

Asym Chi-square test of SIR directions:
                Chisq df   p.value
D=0 vs. D>=1 334.811 54 0.000e+00
D=1 vs. D>=2 101.329 40 3.145e-07
D=2 vs. D>=3  25.398 28 6.061e-01
D=3 vs. D>=4  15.597 18 6.207e-01
D=4 vs. D>=5   7.604 10 6.674e-01
D=5 vs. D>=6   2.251  4 6.896e-01
```

Figure 1: One example of the print-out of the double splicing result.

normal distribution. Life time is generated as $Y^0 = 4 - (|x_1 - 1|) + \epsilon_1$ and censored time is generated as $C = 3 + \epsilon_2$ for $x_1 > 0, x_2 + x_3 > 0$, $C = 10$ otherwise, where $\epsilon_1 \sim N(0, 0.1^2), \epsilon_2 \sim N(0, 0.1^2)$. So life time can only be censored when $x_1 > 0, x_2 + x_3 > 0$. Figure 1 shows the eigen-values and corresponding Chi-square tests of double-slicing. Both the size of the eigen-values and the results of Chi-square tests suggest that the first two eigenvalues are significant. Figure 2 shows the 3D plot of double.slice result, which demonstrates that censored data are clustered in one quadrant. Using the first two SIR directions found in double-slicing to run function censor.sir, only one eigenvalue is significant and the estimated e.d.r. direction of life-time is $(-1.054, -0.003, -0.046, -0.003, -0.012, 0.083)$.

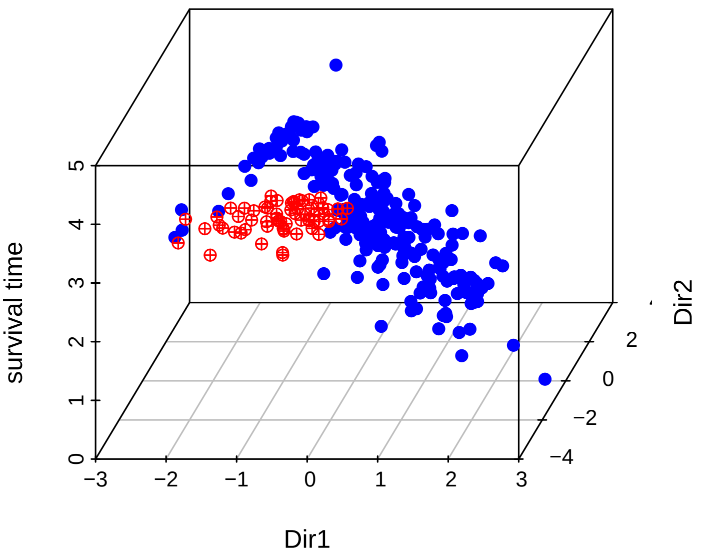


Figure 2: 3D plot of double slicing result. In this example: Dir1=(-1.047, -0.042, -0.076, -0.006, -0.008, 0.100) and Dir2=(0.109, -0.708, -0.731, -0.174, -0.028, -0.099), so Dir1 captures the direction $x_1$ and Dir2 captures the direction $x_2 + x_3$.

# 3 Supplementary results of real data analysis

Please refer to the main text for the outline of this real data analysis. Here we just include the supplementary figures.

```
Double Slicing of Survival time

Number of observation:           295
Number of censored observation:   216
Number of predictors:            22
Number of slices (uncensored):   5
Number of slices (censored):     5

Eigenvalues:
             Dir1   Dir2   Dir3    Dir4   Dir5    Dir6    Dir7    Dir8    Dir9
Eigenvalues 0.2780 0.1631 0.1420 0.09592 0.0825 0.07591 0.05625 0.03682 0.01309
Cum.Sum.R^2 0.2946 0.4675 0.6179 0.71960 0.8070 0.88748 0.94710 0.98612 1.00000
               Dir10     Dir11     Dir12     Dir13     Dir14     Dir15
Eigenvalues 1.956e-15 1.739e-15 1.273e-15 1.114e-15 7.908e-16 5.451e-16
Cum.Sum.R^2 1.000e+00 1.000e+00 1.000e+00 1.000e+00 1.000e+00 1.000e+00
               Dir16     Dir17     Dir18     Dir19     Dir20     Dir21
Eigenvalues 3.948e-16 3.408e-16 3.141e-16 2.701e-16 1.879e-16 9.239e-17
Cum.Sum.R^2 1.000e+00 1.000e+00 1.000e+00 1.000e+00 1.000e+00 1.000e+00
               Dir22
Eigenvalues 7.097e-17
Cum.Sum.R^2 1.000e+00

Asym Chi-square test of SIR directions:
              Chisq  df   p.value
D=0 vs. D>=1 278.360 198 0.0001441
D=1 vs. D>=2 196.355 168 0.0663665
D=2 vs. D>=3 148.229 140 0.3007977
D=3 vs. D>=4 106.348 114 0.6822927
D=4 vs. D>=5  78.052  90 0.8113858
D=5 vs. D>=6  53.715  68 0.8970313
D=6 vs. D>=7  31.321  48 0.9701067
D=7 vs. D>=8  14.726  30 0.9912010
D=8 vs. D>=9   3.862  14 0.9962389
```

Figure 3: SIR output of double slicing step for the 22 genes selected based on correlation and liquid association.

```
Censored Sliced Inverse Regression Model

Number of observation:            295
Number of censored observation:   216
Number of predictors:             22
Number of slices:                 10
Kernel width:                     0.508201095389140

Eigenvalues:
              Dir1   Dir2   Dir3   Dir4    Dir5    Dir6    Dir7    Dir8    Dir9
Eigenvalues 0.3107 0.1906 0.1549 0.1072 0.06287 0.05264 0.03824 0.02848 0.01152
Cum.Sum.R^2 0.3246 0.5238 0.6856 0.7976 0.86326 0.91826 0.95821 0.98797 1.00000
                Dir10     Dir11     Dir12     Dir13     Dir14     Dir15
Eigenvalues 4.344e-16 3.046e-16 2.508e-16 2.295e-16 1.402e-16 1.175e-16
Cum.Sum.R^2 1.000e+00 1.000e+00 1.000e+00 1.000e+00 1.000e+00 1.000e+00
                Dir16     Dir17    Dir18     Dir19     Dir20     Dir21
Eigenvalues 8.389e-17 5.814e-17 3.88e-17 2.684e-17 1.642e-17 1.323e-17
Cum.Sum.R^2 1.000e+00 1.000e+00 1.00e+00 1.000e+00 1.000e+00 1.000e+00
                Dir22
Eigenvalues 1.531e-18
Cum.Sum.R^2 1.000e+00

Asym Chi-square test of SIR directions:
               Chisq  df   p.value
D=0 vs. D>=1 282.362 198 7.662e-05
D=1 vs. D>=2 190.703 168 1.107e-01
D=2 vs. D>=3 134.467 140 6.161e-01
D=3 vs. D>=4  88.770 114 9.615e-01
D=4 vs. D>=5  57.158  90 9.973e-01
D=5 vs. D>=6  38.611  68 9.984e-01
D=6 vs. D>=7  23.081  48 9.991e-01
D=7 vs. D>=8  11.800  30 9.988e-01
D=8 vs. D>=9   3.398  14 9.981e-01
```

Figure 4: SIR output of life e.d.r space recovery step for the 22 genes selected based on correlation and liquid association.

```
Double Slicing of Survival time

Number of observation:             295
Number of censored observation:    216
Number of predictors:              17
Number of slices (uncensored):     5
Number of slices (censored):       5

Eigenvalues:
               Dir1   Dir2    Dir3    Dir4   Dir5    Dir6    Dir7    Dir8
Eigenvalues 0.2141 0.1070 0.07814 0.05983 0.0552 0.03215 0.02294 0.01505
Cum.Sum.R^2 0.3569 0.5354 0.66565 0.76540 0.8574 0.91104 0.94928 0.97438
                 Dir9    Dir10    Dir11    Dir12     Dir13    Dir14     Dir15
Eigenvalues 0.006867 0.002283 0.002161 0.001834 0.0009186 0.000671 0.0004513
Cum.Sum.R^2 0.985830 0.989637 0.993239 0.996297 0.9978283 0.998947 0.9996996
                Dir16     Dir17
Eigenvalues 0.0001615 1.867e-05
Cum.Sum.R^2 0.9999689 1.000e+00

Asym Chi-square test of SIR directions:
                Chisq  df p.value
D=0 vs. D>=1 176.930 153  0.0901
D=1 vs. D>=2 113.781 128  0.8111
D=2 vs. D>=3  82.207 105  0.9512
D=3 vs. D>=4  59.156  84  0.9819
D=4 vs. D>=5  41.508  65  0.9898
D=5 vs. D>=6  25.223  48  0.9972
D=6 vs. D>=7  15.740  33  0.9952
D=7 vs. D>=8   8.973  20  0.9832
D=8 vs. D>=9   4.533   9  0.8730
```

Figure 5: SIR output of double slicing step for the 17 candidate genes, 6 selected based on LA scores and 11 selected based on correlation in the permuted data.

```
Censored Sliced Inverse Regression Model

Number of observation:            295
Number of censored observation:   216
Number of predictors:             17
Number of slices:                 10
Kernel width:                     0.508201095389140

Eigenvalues:
               Dir1   Dir2    Dir3    Dir4    Dir5    Dir6    Dir7    Dir8
Eigenvalues 0.1857 0.1117 0.07176 0.05084 0.03176 0.02253 0.02217 0.01739
Cum.Sum.R^2 0.3546 0.5679 0.70497 0.80207 0.86272 0.90575 0.94809 0.98129
                 Dir9     Dir10     Dir11     Dir12    Dir13   Dir14     Dir15
Eigenvalues 0.009796 2.019e-17 1.819e-17 1.347e-17 9.42e-18 6.9e-18 3.797e-18
Cum.Sum.R^2 1.000000 1.000e+00 1.000e+00 1.000e+00 1.00e+00 1.0e+00 1.000e+00
                Dir16     Dir17
Eigenvalues 2.199e-18 8.746e-19
Cum.Sum.R^2 1.000e+00 1.000e+00

Asym Chi-square test of SIR directions:
                Chisq  df p.value
D=0 vs. D>=1 154.468 153  0.4515
D=1 vs. D>=2  99.694 128  0.9697
D=2 vs. D>=3  66.743 105  0.9987
D=3 vs. D>=4  45.573  84  0.9998
D=4 vs. D>=5  30.574  65  0.9999
D=5 vs. D>=6  21.205  48  0.9997
D=6 vs. D>=7  14.558  33  0.9977
D=7 vs. D>=8   8.019  20  0.9917
D=8 vs. D>=9   2.890   9  0.9685
```

Figure 6: SIR output of life e.d.r space recovery step for the 17 candidate genes in the permuted data. The test of the hypothesis that there is at least one effective dimension reduction (e.d.r) direction of SIR yields an insignificant p-value of 0.45.

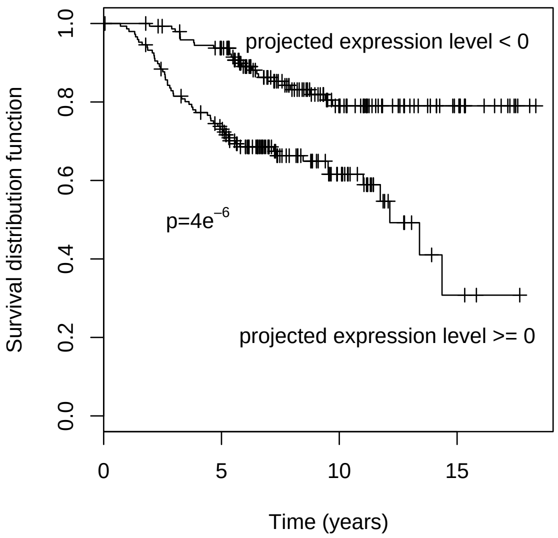


Figure 7: Log-rank test for the permuted data. The patients are divided into two groups of sizes 148 and 147 based on a gene expression signature generated from the permuted data. The log-rank test comparing the two survival curves gives a p-value of $6e^{-6}$. This artifact is largely due to the smallness of the sample size which leads to the chance of overfitting in the permuted data, a phenomenon similar to the one commonly faced in multiple testing without adjustment.

## References

[1] Li KC, Wang JL, Chen CH: **Dimension reduction for censored regression data**. *The Annals of Statistics* 1999, **27**:1–23.
